# Metabolic Processes and Biological Macromolecules Defined the Positive Effects of Protein-Rich Biostimulants on Sugar Beet Plant Development

**DOI:** 10.3390/ijms24119720

**Published:** 2023-06-03

**Authors:** Okanlawon L. Jolayemi, Ali H. Malik, Ramesh R. Vetukuri, Ganapathi V. Saripella, Pruthvi B. Kalyandurg, Tobias Ekblad, Jean W. H. Yong, Marie E. Olsson, Eva Johansson

**Affiliations:** 1 Department of Plant Breeding, Swedish University of Agricultural Sciences (SLU), SE-234 22 Lomma, Sweden; jolayemi.olalekan@slu.se (O.L.J.); ramesh.vetukuri@slu.se (R.R.V.); ganapathi.varma.saripella@slu.se (G.V.S.); pruthvi.balachandra@slu.se (P.B.K.); marie.olsson@slu.se (M.E.O.); 2 Nelson Seed Development AB, SE-223 63 Lund, Sweden; ali.malik@nelsonseed.se; 3 Nelson Garden AB, SE-362 31 Tingsryd, Sweden; 4 DLF Beet Seed AB, SE-261 91 Landskrona, Sweden; tobias.ekblad@hemocue.se; 5 Department of Biosystems and Technology, Swedish University of Agricultural Sciences (SLU), SE-234 22 Lomma, Sweden; jean.yong@slu.se

**Keywords:** agro-wastes, protein-based biostimulants, hydrolyzed wheat gluten, potato protein, sugar beet, growth, physiology and transcriptomic analysis

## Abstract

Protein-based biostimulants (PBBs) have a positive effect on plant development, although the biological background for this effect is not well understood. Here, hydrolyzed wheat gluten (HWG) and potato protein film (PF) in two levels (1 and 2 g/kg soil) and in two different soils (low and high nutrient; LNC and HNC) were used as PBBs. The effect of these PBBs on agronomic traits, sugars, protein, and peptides, as well as metabolic processes, were evaluated on sugar beet in comparison with no treatment (control) and treatment with nutrient solution (NS). The results showed a significant growth enhancement of the plants using HWG and PF across the two soils. Sucrose and total sugar content in the roots were high in NS-treated plants and correlated to root growth in HNC soil. Traits related to protein composition, including nitrogen, peptide, and RuBisCO contents, were enhanced in PBB-treated plants (mostly for HWG and PF at 2 g/kg soil) by 100% and >250% in HNC and LNC, respectively, compared to control. The transcriptomic analysis revealed that genes associated with ribosomes and photosynthesis were upregulated in the leaf samples of plants treated with either HWG or PP compared to the control. Furthermore, genes associated with the biosynthesis of secondary metabolites were largely down-regulated in root samples of HWG or PF-treated plants. Thus, the PBBs enhanced protein-related traits in the plants through a higher transcription rate of genes related to protein- and photosynthesis, which resulted in increased plant growth, especially when added in certain amounts (2 g/kg soil). However, sucrose accumulation in the roots of sugar beet seemed to be related to the easy availability of nitrogen.

## 1. Introduction

Biostimulants are described as bioactive substances that are either organic, inorganic, or microorganisms, which can improve crop performance when applied in small quantities [1,2,3]. Because biostimulants are able to enhance the growth and performance of crops [1,4], increased attention has been seen recently in utilizing them in agricultural and horticultural applications and productions. Reports on biostimulants have indicated their positive impact on crop performance in terms of significant increases in growth and metabolic processes, resulting in increased yield, nutrient- and water-use efficiencies, and tolerance to abiotic stresses [2,4,5,6,7]. Of the different categories of biostimulants, protein-based biostimulants (PBBs), which are also known as protein hydrolysates and amino acids, have received increased interest lately [5]. The PBBs are normally highly available due to the abundance of their raw materials, and thereby the cost of assessing the raw materials and final product is often reasonable [8,9]. The raw materials utilized for the production of PBBs are usually protein-rich wastes, which are generated from agro-allied industries [10]. In some cases, such agro-wastes may also need to find other routes of use, to not end up being dumped into rivers or used as landfills, thereby contributing to environmental pollution [11]. Possible alternative uses of protein-rich residuals from agro-allied industries are in applications in material sciences [12,13] and bioenergy production [11], while they might also be developed into PBBs. Bio-based uses of protein-rich residues from agro-allied industries hold opportunities to result in eco-friendly and sustainable solutions [14], although their economic and environmental effects always need to be properly evaluated [15,16,17,18]. 

As described above, PBBs are often derived through the process of hydrolysis of protein-rich agro-wastes [8,19]. This hydrolysis process (chemical, thermal, enzymatic, or a combination of any of them) contributes to the breakdown of large protein molecules into smaller and more soluble entities [19,20]. The hydrolysis process eventually leads to a mixture of different types of molecules, including peptides and amino acids [8], which are then the main active ingredients in the PBB products [21,22]. Thus, when evaluating the effects of PBBs, it is important to understand effects based on an increased level of N available for the plants, derived from the peptides and amino acids, and of biostimulating effects of other origins [5].

Recent studies have, to an increasing degree, tried to understand the background of the biostimulating effects of PBBs on growth and physiological improvement in crops [23,24,25]. However, despite the fact that measurements of changes in metabolic processes are required to understand the background effects of PBBs, most studies till now have focused mainly on physiological changes [5]. Two PBBs that have been reported to have a biostimulating potential are hydrolyzed wheat gluten (HWG) and potato protein film (PF) [5]. These are both protein-rich streams from the wheat and potato starch industry, respectively [13]. Currently, as for most PBBs, there are no data on metabolic responses to support the physiological effect of HWG and PF on crop growth. Consequently, to improve the understanding of the effects of the use of PBBs and their biostimulating effect, their mode of action in terms of metabolic changes needs to be further evaluated and characterized.

Thus, the aim of the present study was to evaluate the effects of PBBs, i.e., HWG and PF, on the growth and physiological traits of sugar beet. Further, the aim was also to connect the changes in growth and physiological traits to changes in protein and sugar content and composition in the plants and metabolic responses through transcriptomic analysis. 

## 2. Results

### 2.1. Effect of Treatments on Agronomic and Physiological Parameters

**Figure 1:**
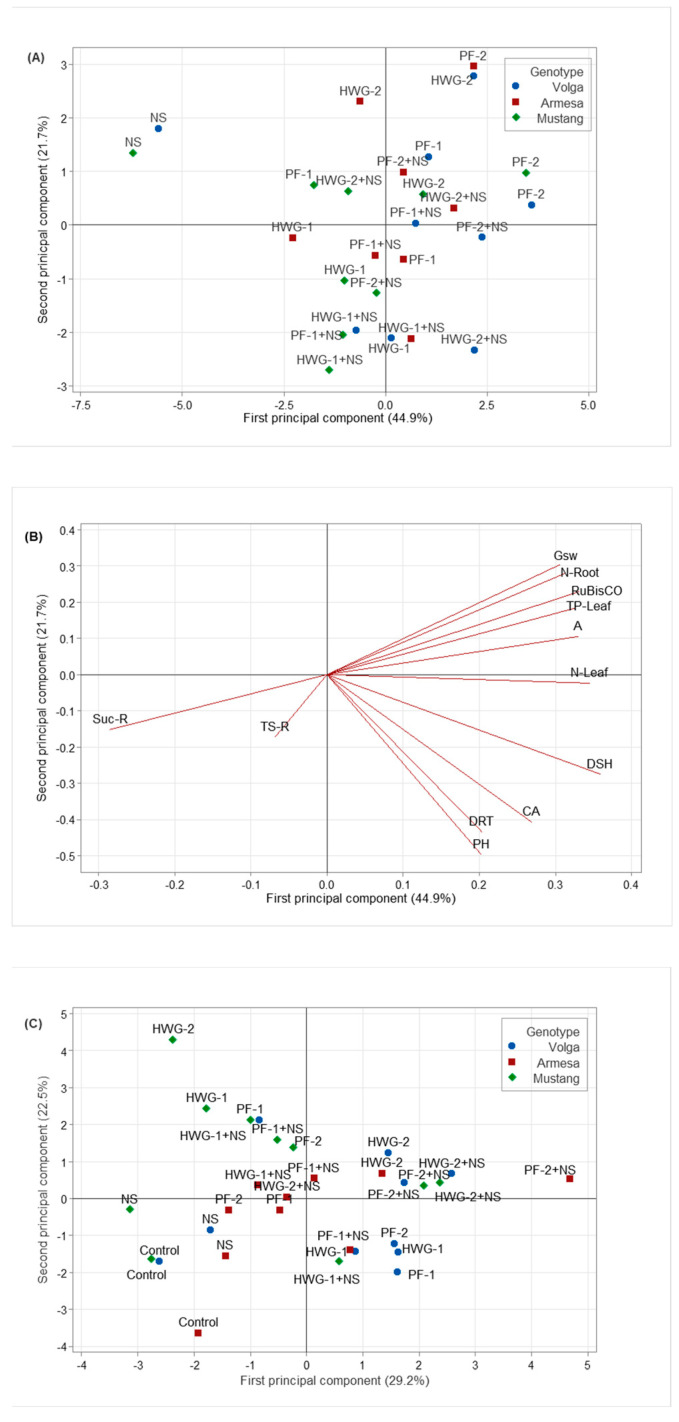

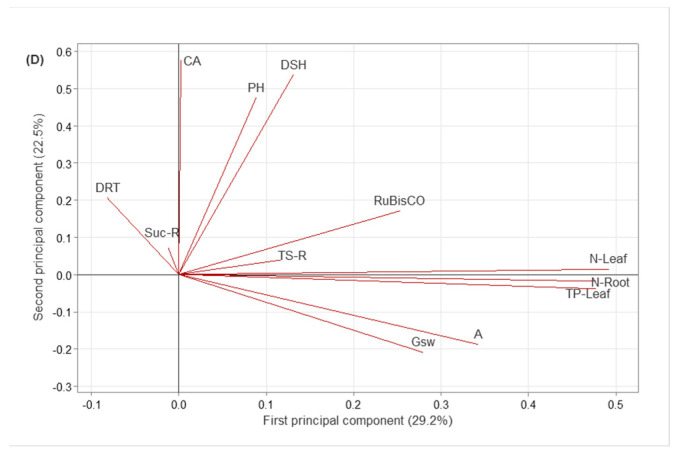
Principal component analysis of all agronomic and physiological parameters of three sugar beet genotypes under PBB and/or nutrient solution treatments. (**A**,**C**) Score and (**B**,**D**) loading plot from PCA of treatments under (**A**,**B**) low nutrient-rich soil conditions (LNC) and (**C**,**D**) high nutrient-rich soil conditions (HNC). PH: plant height, CA: canopy area, A: photosynthetic carbon assimilation rate, Gsw: stomatal conductance, DSH: dry shoot mass, DRT: dry root mass, TP: total peptide in leaf, NLeaf: nitrogen content in leaf, N-Root: nitrogen content in root, Suc-R: sucrose content in root, TS–R: total sugar content in root.

At low nutrient soil conditions (LNC), the samples treated with only nutrient solution (NS) were differentiated from the other samples by principal component analysis (PCA). The NS treatment is located on the negative axis of the first principal component (PC1; Figure 1A), indicating high sucrose and total sugar content in the roots (factors with a negative PCA value) and low values on the other parameters (factors with a positive value; Figure 1B). The high sugar and sucrose content in the NS samples at LNC and low values on the other parameters was also verified by mean values differentiated by Tukey’s posthoc test (Table 1). No clear differentiation was observed based on the rest of the treatments (Figure 1), which was also verified by a large variation in early plant growth influenced by the different biostimulant treatments (Figure 1B, Table S1). However, the control and NS treatment generally resulted in the least plant growth for the three evaluated genotypes of sugar beet (Table S1).

Additionally, at high nutrient soil conditions (HNC), control and NS samples were differentiated (with negative PC1 and PC2 values) from the biostimulant treated samples by PCA (Figure 1C), indicating low values on all the parameters analyzed (Figure 1D). Additionally, these results corresponded with an increased physiological plant development of plants treated with biostimulants (Table S1). Similarly, as for the LNC, the best biostimulant treatments varied in relation to sugar beet genotypes and plant character evaluated (Figure 1, Table S1). 

### 2.2. Impact of PBB Treatments on Photosynthesis and Content of Nitrogen, Peptide, RuBisCO, and Sugar in Roots and Leaves 

The photosynthesis capacity of sugar beet, measured as photosynthetic carbon assimilation, stomata conductance, and chlorophyll fluorescence, was generally low in control and NS samples (Table S1). High nitrogen content in leaves and roots was mostly found in HWG–2+NS, PF–2, and PF–2+NS samples for all three genotypes and under both LNC and HNC, although with some variation (Table 1). The nitrogen content in roots and leaves was generally low in control and NS samples (Table 1). The total peptide and RuBisCO content in the leaves were generally high in the HWG–2+NS, PF–2, and PF–2+NS samples and low in control and NS samples for the three genotypes and under both cultivation conditions, although with the exception of high total peptide content in Volga and Mustang under NS at HNC (Table 1). Differently from the N-related compounds, the sugar contents in the root, especially the sucrose content, were high in NS samples of all three genotypes under both growing conditions (Table 1).

The Pearson correlation analysis revealed a higher degree of correlation between different parameters for LNC than for HNC (Table 2). In principle, a positive and significant correlation was found among all photosynthetic, agronomic, and nitrogen-related parameters for LNC (Table 2). However, sucrose and total sugar content correlated significantly and negatively with other measured parameters (Table 2). For HNC, significant and positive correlations were found among some agronomic parameters (PH, CA, DSH, and DRT), as well as among photosynthetic parameters (A, Gsw) and some of the nitrogen-related parameters (N-L, N-R, and TPL) (Table 2). In HNC, we also observed significant and positive Pearson correlations between Suc-R, DRT, and RuBisCO (Table 2). 

### 2.3. Impact of Amount of N on Agronomic and Physiological Performances

**Figure 2:**
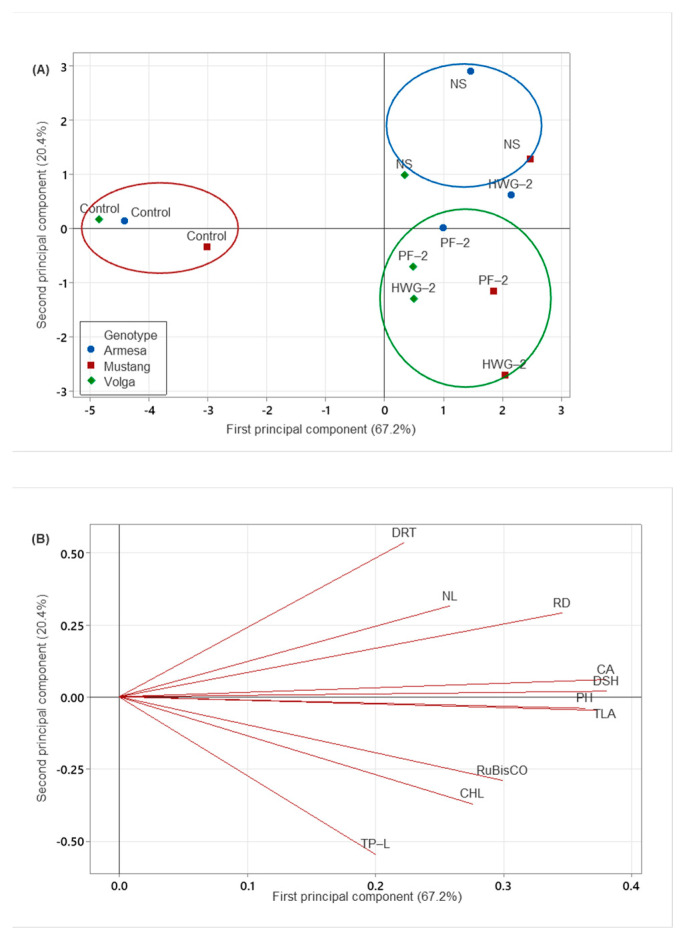
(**A**) Score plot and (**B**) loading plot from PCA of agronomic and physiological parameters of three sugar beet genotypes under equal nitrogen treatment from PBB and NS. CHL: chlorophyll concentration, PH: plant height, CA: canopy area, TLA: total leaf area, DSH: shoot dry mass, RD: root diameter, NL: number of leaves, DRT: dry root mass, TP–L: total peptide in leaf, RuBisCO: content of Ribulose-1,5-bisphosphate carboxylase/oxygenase. Treatments in blue (NS) and green (HWG–2 and PF–2) circles promoted growth parameters and physiological parameters respectively better than treatment in red (Control).

**Figure 3:**
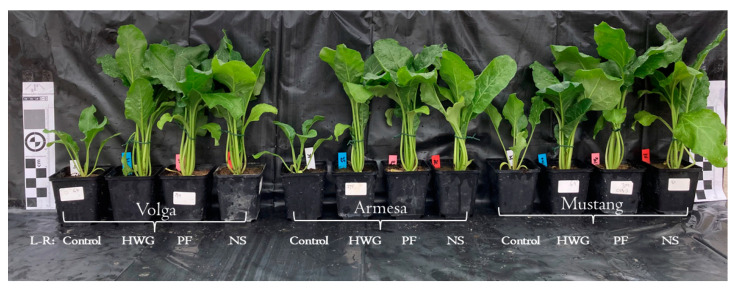
Plant height of three sugar beet genotypes under equal N from HWG, PF, and NS eight weeks after planting.

As the above-evaluated PBBs and NS differed in total N content, an extra experiment was performed, adding them in ratios so that the plants received an equal amount of N from them under HNC, to further reveal their biostimulating effects (Figure 2). As the purpose was to compare effects from different treatments holding the same N content, no PBBs+NS treatments were carried out. The score plot of the PCA differentiated the samples along PC1 based on the amount of N added (control samples with lower N aligned on the negative axis of PC1, while PBB and NS samples with an equal amount of N aligned on the positive axis of PC1) (Figure 2A). This indicated that an increase in nitrogen content as a result of PBB and NS treatments enhanced all agronomic and physiological parameters of sugar beet (Figure 2A,B, and Figure 3). Treatment types (NS versus biostimulants) were differentiated along PC2, explaining 20% of the variation (Figure 2A). Thus, the score plot revealed that the NS treatment (blue circle) favored root traits and the number of leaves (Figure 2B), while PBB treatments enhanced above-ground traits as well as physiological parameters (Figure 2A,B).

### 2.4. Differential Expression of Genes in Leaf and Root Samples of Sugar Beet Treated with PBBs 

**Figure 4:**
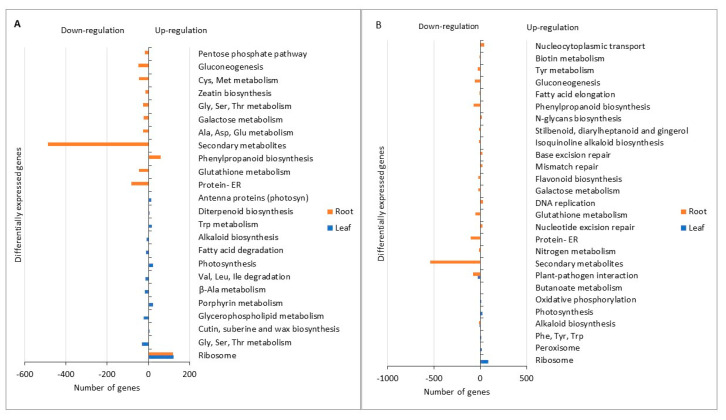
Metabolic processes determined by transcriptomic analysis showing differentially expressed genes from sugar beet leaf and root samples under (**A**) HWG and (**B**) PF treatment. Number of genes with negative values correspond to down-refulated genes, while number of genes with positive values correspond to up-regulated genesthat are relatd to the metabolic processes. Cys: Cysteine, Met: Methionine, Gly: Glycine, Ser: Serine, Thr: Threonine, Ala: Alanine, Asp: Aspartic acid, Glu: Gluatamic acid, Trp: Trptophan, Val: Valine, Leu: Leucine, Ile: Isoleucine, ER: Endoplasmic reticulum, Phe: Phenylalanine, Tyr: Tyrosine.

The transcriptomic analysis carried out based on the use of PBBs on sugar beet revealed large differential gene expressions. In total, 14,000–16,000 genes in both leaf and root samples were differently expressed (DE) compared to the control as a result of the PBB treatments (Table 3). The change in the expression of genes resulted in either an up- or a down-regulation of genes, and the present study clearly showed that a higher number of genes were down- than upregulated by the PBBs, especially in the root (Table 3). Furthermore, the change in expression of genes was generally higher in the roots as compared to in the leaves. Although the use of HWG resulted in a greater change in the expression of genes in the leaves than the use of PF, the opposite was found in the roots, where a greater change was obtained for PF than for HWG (Table 3, Figure 4).

The down-regulation in the roots of genes associated with the biosynthesis of secondary metabolites was the most obvious change for both PBBs used as compared to the control, accounting for 50–60% of the down-regulated genes (Figure 4). Other genes that were down-regulated for both PBBs were some genes associated with specific secondary metabolites (tropane, piperidine, and pyridine) and genes associated with glutathione and galactose metabolisms.

Furthermore, when HWG was used, a down-regulation was found of genes associated with biosynthesis or metabolism of some amino acids (glycine, serine, threonine, valine, leucine, isoleucine, beta-alanine, aspartate, glutamate, cysteine, methionine, and zeatin), and of some fatty acid and respiration-related genes (Figure 4a). For PF, additional genes down-regulated in the roots included those involved in protein processing in the endoplasmic reticulum and plant-pathogen interaction genes (Figure 4b).

The most clearly upregulated genes were those associated with the ribosomes in both leaves and roots for the HWG-treated plants, while only in the leaves for the PF-treated plants (Figure 4). Additional upregulated genes for both HWG- and PF-treated plants were those for photosynthesis (Figure 4). Further, genes associated with cutin, suberin, and wax biosynthesis, tryptophan metabolism, and diterpenoid biosynthesis in the leaves and phenylpropanoid biosynthesis in the roots were upregulated for HWG-treated plants (Figure 4a). However, genes associated with the biosynthesis of aromatic essential amino acids (phenylalanine, tyrosine, and tryptophan), peroxisome as well as butanoate metabolism were upregulated in the leaves, and genes associated with DNA replication, mismatch repair, nucleotide excision repair, N-glycan biosynthesis, and base excision repair were upregulated in the roots of PF-treated plants (Figure 4b).

To validate the NGS results, the relative expression levels of five randomly selected genes were tested using a quantitative reverse transcription PCR (qRT-PCR). To that end, we selected three genes that are statistically significantly DE in all four treatments, *BvHSP70* (Gene ID 104883827), which encodes a chloroplast membrane-associated heat shock protein *BvHIPP24* (Gene ID 104905684), that codes for a heavy metal-associated isoprenylated plant protein, *BvGR2*(Gene ID 109135315), encoding glutamate receptor 2.6-like protein. Additionally, we validated the expression of, *BvIAA6* (Gene ID 104904637)*,* which encode auxin-induced protein IAA6, and *BvSUSIBA2* (Gene ID 104890228)*,* encoding a WRKY transcription factor that is involved in sugar signaling. The results indicated that the expression levels obtained from qRT-PCR analysis were largely consistent with the NGS data (Figure S1). However, the relative expression of *BvHIPP24* and *BvGR2* were upregulated in root samples of the plants treated with HWG according to the qRT-PCR as opposed to downregulated in the NGS data. Notably, the analysis indicated a positive correlation (correlation coefficient, r = 0.70) between the log2 fold change obtained from the NGS and qRT-PCR data. Interestingly, the correlation coefficient was above 0.90 for leaf samples treated with HWG and PF and root samples treated with PF, indicating a strong positive correlation between NGS and qRT-PCR data (Figure S1).

## 3. Discussion

The present study clearly showed that the used PBBs had a biostimulating effect on sugar beet. Thus, these PBBs contributed, when applied in specific amounts (2 g/kg soil), to up-and down-regulation of certain genes, resulting in an increased protein- and photosynthesis. These changes in gene activities resulted in an increase in the content of all nitrogen-related parameters in the leaves and thereby led to increased plant growth. However, in soils with HNC, nutrient solution (NS) with easily available nitrogen contributed specifically to increased root growth in the young sugar beet plants. 

The present study showed that the enhancement of nitrogen-related parameters (nitrogen content, total peptide, and RuBisCO content) by the use of PBBs was a reflection of the activation of ribosome genes, as highlighted by the transcriptomic results. Nitrogen, peptide, and RuBisCO are N-rich molecules that are precursors for protein synthesis, which may have contributed to the increased ribosome activity, as the ribosome is an organelle for protein synthesis [26]. However, in roots treated with HWG or PF, genes associated with protein processing in the endoplasmic reticulum were down-regulated. This indicates, as also suggested by previous studies [27], that molecules such as peptides and amino acids are mobilized in the roots for further transport to the leaves, where protein synthesis takes place. Such a mechanism was also further verified here and in previous studies [28] by the high nitrogen and peptide content observed in leaf samples as compared to root samples treated with PBBs. Additionally, corresponding to previous studies [29], genes associated with photosynthesis were upregulated in the leaf samples of plants treated with either HWG or PF compared to the control. Such an up-regulation contributes to the enhancement of photosynthetic carbon assimilation and RuBisCO accumulation, as this enzyme is known to be highly involved in the process of photosynthesis [30]. Furthermore, genes associated with glycolysis and galactose metabolism, which are related to the respiration process, were down-regulated in the roots of samples treated with either HWG or PF. This down-regulation might be a result of the storage of sugar in sugar beet roots. It is well known that respiration is a catabolic process that results in the breakdown of sugars or other respiration substrates in order to release energy and carbon dioxide [31]. Thus, a down-regulation of the metabolic processes in the sugar beet roots may prevent respiration, thereby enhancing sugar storage in the root of the sugar beet plant. 

The present study showed that RuBisCO at equal nitrogen additions, from the use of both NS and PBBs, were found to have a positive impact on sugar beet agronomy and physiology. However, the PBBs (both HWG and PF) enhanced all nitrogen-related parameters of sugar beet, including nitrogen content in leaf and root, and total polypeptide as well as RuBisCO content under both LNC and HNC as compared to control and NS, while NS was shown to favor root growth. RuBisCO in the leaves and the root dry mass were positively and significantly correlated to sucrose content in the roots at HNC. These results indicated differences in the mode of action of the NS and PBBs on sugar beet growth and physiology. The active ingredients in PBBs, similar to other protein hydrolysates, are mixtures of peptides and amino acids [2,5,24,32,33,34], unlike nitrate and other mineral elements present in NS. These active ingredients are usually low molecular weight compounds that are easily taken up by plant roots or foliage and which can act as precursors for phytohormones that are responsible for growth and development or nutrient sources used directly for growth [33]. This corresponds with the findings of the present study, indicating different pathways of plant growth and development in relation to used sources of nitrogen (NS versus PBBs).

In addition to the up-regulation of genes related to ribosomes and photosynthesis, genes for aromatic amino acids, e.g., tryptophan, were also upregulated. These aromatic amino acids (phenylalanine, tyrosine, and tryptophan) are known as precursors for auxins biosynthesis and other plant secondary metabolites, which enhance plant growth and tolerance to environmental stresses [35,36]. Furthermore, the HWG treatment resulted in an up-regulation of cutin, suberin, and wax biosynthesis as well as of diterpenoid biosynthesis in leaf samples. Cutin and waxes are known as water-resistant fatty acids derivatives, which are deposited on different parts of the plant, especially the leaves, and they provide minimum resistance to microbial penetration through leaf surfaces [37]. Previous studies have shown that diterpenoids are connected to the formation of gibberellin (GA), a phytohormone responsible for apical growth [29]. The HWG treatment also resulted in an up-regulation in the root of phenylpropanoid genes, which in previous studies have been linked to enhanced capacity to prevent microbial infection [38]. This is because phenylpropanoids (also known as cinnamic acids) help to induce a response to fungal infections [39]. Furthermore, genes related to base excision repairs, nucleotide excision repair, mismatch repair, DNA replication, and nucleocytoplasmic transport were upregulated in the roots of the plants treated with PF, traits that previously had been linked to stress tolerance [40]. The differences in metabolic processes resulting from the differential gene expression by the use of HWG and PF confirmed that both PBBs are composed of different active ingredients (peptides and amino acids). Previous studies have indicated that differences in active ingredients are affected by the hydrolysis or other processes of making the PBBs [21].

The enhanced agronomic performance of plants treated with PBBs corresponds with the results of previous studies, primarily using protein hydrolysates but, in some cases, amino acids on a range of different plant types [14,20,21,33,34,41,42,43]. Additionally, small molecules such as polyamines have been reported to improve growth and stress tolerance ability in plants [44]. Previous studies have also reported positive physiological responses in terms of photosynthetic parameters in different crops from the use of PBBs [45,46], which our results verified. Additionally, the high sucrose content obtained in the present study in roots of sugar beet treated with HWG, PF, or NS, either individually or in combination, corresponded well with results from previous studies [43]. However, such a combination did not prove to be spectacular over solely applied PBBs. Furthermore, from the present study, no clear genotype differences were seen as to their performance in relation to the used PBB in regard to their agronomic and physiological parameters.

## 4. Materials and Methods

### 4.1. Plant Materials and Protein-Based Biostimulants (PBB)

Hybrid seeds of three sugar beet genotypes (Volga, Armesa, and Mustang) were generously provided by DLF Beet Seed AB, Landskrona, Sweden. These genotypes were selected because they represent the sugar beet gene pool in both the Nordic region and the EU. Two protein-based biostimulants (HWG and PF) were used in this study. Wheat gluten (WG) and potato protein (PP) are available as side streams from the industry [5]. In the present manuscript, we used the hydrolyzed WG (=HWG) as it is supposed to have better performance as a protein-rich biostimulant when the polymerized structure of the proteins is broken [5]. We preferred for this experiment not to produce the HWG by ourselves as it is easily purchased. HWG was purchased at A. Constanstino & Co. S.P.A., Favria, Turin, Italy. The PF was produced by ourselves in the lab from the PP received from the industry. We preferred to use PF and not PP based on previous results [5]. The PF was a film cast from potato protein powder, which was generously supplied by Lyckeby, Kristianstad, Sweden. PF was made by dispersing 50 g potato protein powder in 500 mL of milli-Q water over a 5 mm sieve. The suspension was placed on a magnetic stirrer for 10 min at 500 rpm at room temperature. The suspension was then dispensed into 100 mm × 15 mm Petri dishes at a volume of 50 mL per Petri dish. The dispensed suspension was placed in an oven for 48 h at 45 °C in order to form dry friable flakes (film) [47]. 

### 4.2. Soil Types and Biostimulants Treatments

Two trials were conducted in this study based on the soil types used. Soil type A is basically composed of sand, which was supplied by Bara Mineraler, Malmo, Sweden. This soil type (sandy) was chosen in order to be able to evaluate the effect of PBB in nutrient-deficient soil and for maximum root extraction for root biomass analysis. Whereas soil type B is a mixture of soil type A (sand) and peat-based soil in a ratio of 3:1. Peat-based soil was supplied by Emmal-Junga Torvmull AB, Sweden, and its physical and chemical components are presented in Table 4a. Soil type B was chosen in order to evaluate the effect of soil with improved physical and chemical characteristics in combination with PBB and/or NS on sugar beet growth and physiology. Both HWG and PF were mixed with the two soil types (A and B) in different concentrations (1 and 2 g/kg soil) either individually or in combination with nutrient solution (NS). NS used in this study was generously provided by DLF Beet Seed AB, Landskrona, Sweden. The NS contained macronutrients and micronutrients in concentrations suitable for the greenhouse cultivation of sugar beet (Table 4b). The NS treatment used in the present study was 25 mL NS per plant at four weeks after planting, without the addition of any PBB. Ten replications of the treatments per variety were generated, and details are presented in Table 5. The choice of the concentrations (1 and 2 g/kg soil) was made based on the results from the previous study [5]. The untreated soil (no PBBs and/or no NS) was maintained as a control. 

### 4.3. Design of Experiment and Environmental Conditions

The experiments were set up in the growth chamber of the Biotron at the Swedish University of Agricultural Sciences (SLU), Alnarp, in a controlled environment. The experiments were laid out in a completely randomized design in ten replicates. The temperature (3.0/10.0 °C), relative humidity (60–70%), day length (13/11 h), and light intensities (0–1000 µmol m^−2^ s^−1^) were set to mimic the weather condition during sugar beet planting season (March/April) in Southern Sweden. After four weeks in the Biotron, the experiment was moved to a greenhouse with a controlled relative humidity of 80–90% and temperature of 12–15 °C. The day length and light intensity in the greenhouse corresponded with the prevailing weather during the experiment, which was between March and August.

### 4.4. Effects of Equal Nitrogen Content from PBBs and NS on Agronomic and Physiological Parameters of Sugar Beet

In order to understand the mode of action of HWG and PF, an additional experiment was set up using equal amounts of nitrogen from HWG, PF, and NS supplied to the sugar beet genotypes. The nitrogen content (~13%) present in the HWG and PF was estimated using the Dumas method (Flash 2000 NC Analyzer, Thermo Scientific, Waltham, MA, USA, NX6.25) (more details under nitrogen content analysis). Thus, the addition of 2 g/kg HWG and PF at 2 g/kg as a PBB concentration for optimum growth of sugar beet [5] resulted in a supply of 260 mg N per kg of soil per plant. To contribute the same quantity of N by application of NS, ~960 mL of NS (composition described above) was supplied to the sugar beet plant with NS treatment. 

Plants were raised under similar conditions as described above in both the Biotron and greenhouse for eight weeks. Similarly, agronomic and physiological data were collected at the end of the experiment.

### 4.5. Data Collection

#### 4.5.1. Growth 

Data on the growth of sugar beet were collected at eight weeks after planting (WAP). Growth parameters were measured as previously described [5] and included plant height (cm) and digital plant canopy area (% green pixel/cm^2^). Plant height was measured using a transparent 50-cm meter rule from the topsoil to the tip of the tallest leaf. The plant canopy area was measured by taking aerial view photographs of plants from 60 cm above the plant using a Fuji Film Camera. The pictures were then analyzed using Easy-Leaf-Area (ELA), an open-source software for phenotyping [48]. The percent green pixel in relation to the area of coverage was taken to be the canopy area, but values were presented in squared centimeters (cm^2^). 

#### 4.5.2. Gas Exchange Measurement

Gas exchange and chlorophyll fluorescence were measured on eight-week-old sugar beet seedlings under different PBB treatments. The measurements were taken on fully developed leaves in the morning (09:00–11:00) using a portable and open system equipped with infrared gas analyzers (model 6800; Li-Cor Inc., Lincoln, NE, USA). The leaf temperature during measurements was maintained at 25.0 ± 0.5 °C. Leaves were illuminated with a steady red and blue light source at a photosynthetic photon flux density (PPFD) of 1200 μmol m^−2^ s^−1^ [49]. The reference CO_2_ concentrations in the cuvettes matched the treatment CO_2_ concentrations to which sugar beet plants had been growing, i.e., 400 ± 2.5 μmol mol^−1^. The vapor pressure deficit (VPD) was 1.1 ± 0.05 kPa, and the relative humidity was 55–65%. The gas exchange instrument was calibrated each day before the measurements and matched at least twice a day (between the curves). Data were recorded after sample acclimation in the cuvette for at least 15 min. Data were collected after the prevailing CO_2_ had reached a steady state (2–3 min).

#### 4.5.3. Biomass and Physiological Sample Collection

Plants were carefully uprooted and separated into shoots and roots at 8 WAP for analyses of biomass and physiological parameters (polypeptide and sugar analyses using HPLC as well as nitrogen content using the Dumas method). Shoot and root samples were washed under gentle running tap water to remove soil particles. Excess water from the washed shoot and root samples was drained using a 3-fold paper towel. Three selected plants per treatment were put in separate brown paper envelopes (26 cm by 16.5 cm) and dried at 70 °C for 72 h in a ventilated drying cabinet for biomass analysis. Well-dried plant samples were weighed on a digital scale calibrated in milligrams. Thereafter, two plants, separated into shoot and root, were placed in separate plastic zipper bags and stored in a −80 °C freezer for analysis of physiological parameters. 

#### 4.5.4. Analyses of Biological Macromolecules

##### Sample Preparation and Protein Extraction; Total Polypeptide and RuBisCO Contents Analysis Using Size Exclusion (SE)-HPLC

Two plants per treatment, separated into shoot and root samples, frozen at −80 °C, and later used for RuBisCO, total polypeptide, sucrose, and total sugar content analyses using HPLC, as well as for analyses of nitrogen content using the Dumas method. The frozen (−80 °C) samples were freeze-dried for 72 h and then ground into powder using an MM 400 Retsch ball mill (Retsch Mill. Haan, Germany). Ground samples were put in 50 mL Falcon tubes and kept in a −4 °C freezer until further use.

The protein extraction protocol was similar to Gupta et al. [50], with some modifications. In order to extract protein, 16.5 mg of ground sample (leaf or root) was measured into 1.5 mL micro-centrifuge tubes in three replicates. Phosphate buffer (pH 6.9) prepared from a mixture of 0.05 M NaH_2_PO_4_·H_2_O (Solution A, MW: 137.99) and 0.05 M NaH_2_PO_4_·2H_2_O (Solution B, MW: 177.99) solutions in ratio 1:1, was added to the sample in the tubes at the rate of 1.4 mL per sample. Sample mixtures were vortexed and then placed on a shaker for 5 min at 2000 rpm (IKA Vibrax VXR B, IKA Werke, Germany) for protein extraction. Samples were then arranged in the centrifuge and set at 10,000 rpm for 30 min for protein extraction. After centrifugation, the clear liquid phase above the samples was decanted into 2.0 mL HPLC vials and arranged in the HPLC autosampler.

**Figure 5:**
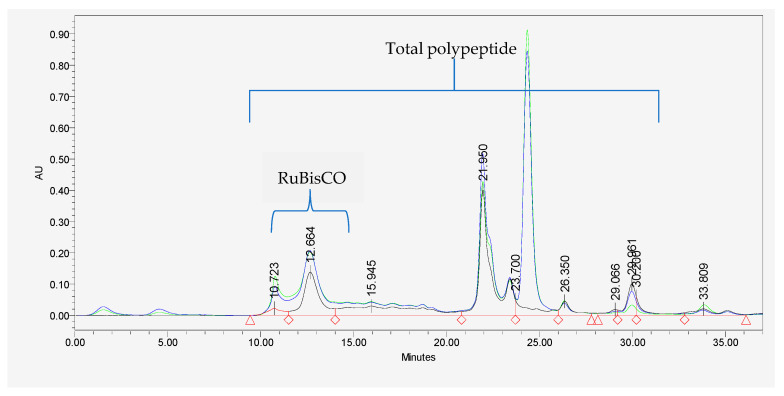
Example of SE-HPLC showing different peaks of protein analysis based on PBB treatment of sugar beet leaf sample. Blue line: HWG treatment; green line: PF treatment; black line: NS treatment; and red line: phosphate buffer. Red rhombus and triangle at the base of the figure are only to align the baseline to the origin (zero). Numbers above the peaks indicated the retention time for each peaks in minutes.

Protein extracts in the HPLC vials were arranged in Waters e2695 HPLC machine with a Waters 2998 PDA detector (Waters Corporation, Milford, MA, USA), fitted with SEC s2000 column (Phenomenex, Torrance, CA, USA), which is suitable for analyzing polypeptides and small proteins (~3 kDa). The method was set to collect 20 µL per sample (at 25 °C), which runs for 37 min, and each treatment is made up of three replicates, while the column (SEC s2000) was maintained at 19 °C. A mobile phase of 0.05 M NaH_2_PO_4_, pH adjusted to 6.9, was applied at 0.5 mL/min. Absorption spectra (3D) were collected at 190 to 520 nm over 37 min, and for further analysis, absorption at 210 and 280 nm were collected. Phosphate buffer (solutions A and B, ratio 1:1, pH 6.9) was used as blank and was set at the end of running each replicate (containing ten treatments). The chromatograph from the SE-HPLC generated by Waters Software (Empower 2) was used to estimate the total peptide and RuBisCO contents present in each treatment by determining the area under the curve. Total peptide content per treatment was estimated by the sum of all the areas under the curves of the chromatogram, measured at 210 nm wavelength (Figure 5). However, RuBisCO content was estimated by calculating the area under the curve at a retention time (RT) of 10.0 min on the chromatogram at 280 nm wavelength.

##### Nitrogen Content Analysis Using the Dumas Method

Approximately 5 mg of ground leaf and root samples of sugar beet treated with PBBs were measured into thin aluminum capsules. The aluminum capsules were then folded and pressed to remove excess air that may be trapped in the capsule. The nitrogen content of PBB-treated sugar beet in leaf and root samples was determined using the Dumas method with a Flash 2000 N/C Analyser (Thermo Scientific, Waltham, MA, USA) [13]. The results of the nitrogen content of the samples were presented as averages from triplicates.

##### Sugar Content Analysis Using HPLC Method

Based on the result of a preliminary experiment, a 75 mg ground root sample was used for the analyses, and one-milliliter milli-Q water was added to each sample, followed by vortexing, shaking for 5 min, and centrifugation at 8000 rpm for 5 min. The supernatant was carefully decanted and diluted five times (100 µL in 500 µL milli-Q water) before running the samples in the HPLC system (Agilent 1100 OpenLab software ChemStation Edition v2.7, Santa Clara, California, USA). The HPLC system was connected to an Agilent 1260 Refractive Index Detector (RID), fitted with Asahipak NH2P 50 4E column, and eluted with 0.8 mL/min 5 mM H_2_SO_4_. Samples were injected into the system at the rate of 10 µL for 10 min and maintained at room temperature, while the RID was maintained at 35 °C. Standard sugar solution containing 2.5% each fructose (Janssen Chimica Geel Belgium) glucose and sucrose, as well as 0.5% raffinose from Sigma Chemical Co., St. Louis, MO, USA, were used to identify the peaks of the different sugars. The formula below was used to estimate the sucrose (%) and total sugar content (%) that is present in the root samples of sugar beet.

$$\begin{array}{c} Sucrose\;content\left(\%\right)\\ \;\;\;\;\;\;\;\;\;\;\;\;\;\;\;\;\;\;\;\;\;\;\;\;\;\;\;\;=\frac{Area\;under\;curve\;by\;sample\;\times \;sucrose\;content\;in\;standard\;\left(2.5\%\right)\;\times \;dilution\;factor\;(5)}{Area\;under\;curve\;by\;sucrose\;standard} \end{array}$$

$$\begin{array}{c} Total\;sugar\;content\left(\%\right)\\ \;\;\;\;\;\;\;\;\;\;\;\;\;\;\;\;\;\;\;\;\;\;\;\;\;\;\;\;=\frac{Area\;under\;curve\;by\;sample\;\times \;total\;sugar\;content\;in\;standard\;\left(8\%\right)\;\times \;dilution\;factor\;(5)}{Area\;under\;curve\;by\;total\;sugar\;standard} \end{array}$$

### 4.6. Data Analysis

All growth, biomass, and physiological parameters were subjected to analysis of variance (ANOVA) using a general linear model (GLM) of Minitab 19.2 in order to detect significant differences in the treatments. Thereafter, means were separated using the Tukey posthoc test, where differences were indicated with different lowercase alphabets. Principal component analysis (PCA) was carried out using all measured parameters (growth, biomass, and physiology) with Minitab 19.2. Correlation analysis was done using the “data analysis plug-in” of Microsoft Excel, and cells were formatted using the color scale tab of Microsoft Excel in order to evaluate the differences in the correlation of parameters. 

### 4.7. Transcriptomics Analyses

#### 4.7.1. RNA Sample Collection, Sequencing, and Data Analysis

Sugar beet genotype Armesa was used for the transcriptomic analysis because it largely represents the gene pool in the Nordic region. Armesa was sown in pots filled with peat-based soil containing sand and peat in a ratio of 1:3 (HNC) treated with 2 g/kg of HWG or PF, and untreated (no HWG and PF) pots were maintained as the control for eight weeks. At eight weeks after planting, leaf and root samples for transcriptomic analysis were collected as three biological replicates; each biological replicate was pooled from three individual plants. Leaf or root samples were collected and snap-frozen in liquid nitrogen and stored at −80 °C. RNA extraction was done following a similar method described by [51]. One hundred milligrams of tissue was homogenized in a motor and pestle in liquid nitrogen, followed by RNA isolation using an RNeasy Mini kit (Qiagen, North Rhine-Westphalia, Germany). Extracted RNA was then treated with a Turbo DNA-free kit (AM1907, Thermo Scientific, Waltham, MA, USA) to remove any genomic DNA contamination. The quality of RNA was assessed on Agilent Bioanalyzer. Paired-end mRNA reads were generated using Illumina high-throughput sequencing from the NGI facility. A quality control (QC) check was performed on independent samples with three biological replicates per sample using the FastQC v0.11.7 tool [52], and multiple sample visualization was evaluated using the MultiQC v1.6 tool [53]. An initial filtering step was performed on the removal of ribosomal RNAs (rRNAs) by aligning reads with silva and Rfam databases using the Sortmerna-v2.1b [54] tool, and all TruSeq3 adapters were trimmed with Trimmomatic-v0.36 [55] setting MINLEN:20 in bases and SLIDINGWINDOW:5:20 with other default parameters. The second round of QC checks was performed using the same tools mentioned above.

The whole genome of Beta vulgaris EL10_1.0 (https://phytozome-next.jgi.doe.gov/info/Bvulgaris_EL10_1_0; accessed on 15 May 2023) was used for reference alignment. The mRNA reads were aligned to the CDS coordinates using the splice aligner STAR-v2.7.5b [56] tool with --twopassMode Basic, --sjdbGTFfeatureExon CDS parameters, keeping other settings as default, the total number of reads processed can be seen in the Supplementary Table S3. Transcript abundance was estimated with Salmon v1.3.0 [57]. Raw read counts were used for Differential Expression (DE) analysis with DESeq2 [26,58], and an in-built cross-sample “Relative Log Expression” (RLE) [27] normalization was performed.

#### 4.7.2. Pathway and GO Terms Enrichment Analysis

Kyoto Encyclopaedia of Genes and Genomes (KEGG) terms pathway enrichment analysis was performed using obtained gene coordinates from the closest variety of Beta vulgaris genome RefBeet-1.2.2 (https://www.ncbi.nlm.nih.gov/assembly/GCF_000511025.2/; accessed on 15 May 2023). Each pairwise comparison of the DE gene set filtered with FDR cut-off < 0.05 was used for Gene Set Enrichment Analysis (GSEA). GSEA of KEGG were tested with the clusterProfiler version 3.18.1 [28] submodule gseKEGG with settings nPerm = 10,000, pvalueCutoff = 0.05; pairwise comparisons are shown in Supplementary Figure S2, and data tables are available in the Supplementary Table S4. 

Singular Enrichment Analysis (SEA) of Gene Ontology (GO) categories was performed on AgriGO v2.0, the web-based tool using mapped coordinates obtained from the PLAZA3.0 database (https://bioinformatics.psb.ugent.be/plaza/versions/plaza_pico_03/; accessed on 15 May 2023). The DE gene set was filtered with both FDR < 0.05 and log2FC > 1.0. GO significance level of FDR < 0.05 was set, and keeping the remaining settings as default, data tables are available in Supplementary Table S5.

#### 4.7.3. qRT-PCR Analysis

qRT-PCR analysis was carried out as described previously [31]. Briefly, 500 ng of total RNA was used for reverse transcription using an iScript cDNA synthesis kit (BioRad, Hercules, CA, USA). qRT-PCR reactions were performed using 2×DyNamo Flash SYBR Green Master mix kit (Thermo Scientific, Waltham, MA, USA) following manufacturer’s instructions with four microlitres of 10-fold diluted cDNA used as a template. Data analysis was performed using the BioRad CFX manager 3.1 with the Cq values of target genes normalized to that of BvGAPDH and BvEF1 alpha genes. The primer sequences are listed in Table S6. All reactions were performed with three biological replicates per treatment. Each biological replicate had three technical replicates for the qRT-PCR.

## 5. Conclusions

Hydrolyzed wheat gluten (HWG) and potato protein film (PF) are two novel protein-based biostimulants (PBBs) from agro-industrial side streams with biostimulating effects on sugar beet plant development. Both HWG and PF enhanced the early growth and development of sugar beet plants as well as the synthesis and/or accumulation of bio-macromolecules such as peptides, RuBisCO, sucrose, and total sugar content. Furthermore, the application of HWG and PF contributed to the up-regulation of genes associated with important metabolic processes such as protein synthesis, photosynthesis, and biosynthesis of metabolites and aromatic amino acids. All of these metabolic processes lead to enhanced growth and physiology of sugar beet, either directly through photosynthesis and protein synthesis or indirectly by the effect of amino acids (auxins and gibberellic acids) for plant growth and development and stress tolerance. Differences in up-regulation of genes, e.g., for DNA replication, mismatch repair, and nucleotide excision repair, in plants treated with HWG and PF indicated variation in the presence of active ingredients (amino acids and peptides) in the two PBBs, resulting in different effects on metabolic processes. The positive effect on the early growth of sugar beet plants and the biostimulating effect from the use of the HWG and PF indicate their possible use in crop production. As these PBBs and their raw materials are obtained from side streams of the agro-industry, they are expected to be environmentally friendly and sustainable, although this must be further verified. 

## Figures and Tables

**Table 1:** Physiological traits of sugar beet under PBB treatments in different soil nutrient conditions.

Genotype	Treatment	Low Nutrient Condition						High Nutrient Condition					
		N-Leaf	N-Root	TP-Leaf	RuBisCO	Suc-R%	TS–R%	N-Leaf	N-Root	TP-Leaf	RuBisCO	Suc-R%	TS–R%
Volga	Control	0.9h	1.0f	43.1e	9.5f	0.5d	1.0e	1.7d	0.8d	61.0g	13.7g	0.2g	3.3e
	HWG–1	1.8e	1.4d	44.8e	30.4e	3.0b	5.9a	3.4a	1.3b	57.7g	38.6d	2.0a	7.6b
	HWG–1+NS	1.4f	0.9f	57.8d	49.9d	3.2b	6.4a	2.4c	0.8d	149.9b	44.4c	0.4e	8.0b
	HWG–2	3.4a	2.7a	52.2e	75.3c	0.4d	3.8d	3.3a	1.2b	84.1f	36.0d	0.2g	9.8a
	HWG–2+NS	2.8b	1.4d	107.4a	85.0b	0.9c	6.4a	3.5a	1.4a	137.7c	52.2b	0.3f	3.9e
	NS	0.7h	1.1e	68.8c	8.0g	3.8a	5.3b	1.6d	1.0c	161.1a	23.5f	1.7b	8.2b
	PF–1	2.0d	2.0c	78.0b	52.4d	0.2e	5.1b	2.8b	1.3ab	126.5d	31.1e	0.6d	6.8c
	PF–1+NS	2.1d	1.9c	81.6b	76.6c	0.5d	5.8a	2.9b	1.3a	107.2e	53.6b	1.6c	5.3d
	PF–2	2.5c	2.5b	108.3a	136.1a	ND	ND	3.2ab	1.4a	137.0c	51.2b	1.8b	5.7d
	PF–2+NS	2.8b	2.9a	118.5a	74.1c	0.3e	4.5c	3.3a	1.3ab	139.6c	67.0a	1.8b	8.2b
Armesa	Control	1.2f	1.0g	33.2d	8.1g	2.2b	3.0d	1.6e	0.7f	52.2e	22.8h	4.6c	4.3c
	HWG–1	1.5e	1.0g	32.8c	11.9f	3.6a	7.1ab	2.8c	1.0d	58.0d	110.1c	4.3c	6.9ab
	HWG–1+NS	2.2d	1.3f	40.7c	12.5f	0.6c	7.7a	2.1d	0.9e	94.7b	121.9b	6.2ab	7.1a
	HWG–2	1.5e	1.0g	54.0b	88.3b	ND	ND	3.1b	1.0d	63.6c	72.2d	6.8a	7.6a
	HWG–2+NS	2.9b	2.2b	64.1a	86.1b	0.3e	4.9c	1.6e	1.0d	93.3b	68.7e	5.6b	6.3b
	NS	0.9g	0.7h	64.9a	3.3h	3.4a	5.3c	2.1d	0.9e	50.5e	70.4d	6.7a	7.3a
	PF–1	2.2d	1.4d	55.6b	43.5d	0.6c	6.5b	2.8c	1.1c	87.1c	69.9de	6.5a	7.3a
	PF–1+NS	2.1d	1.3e	56.5ab	28.9e	0.4d	6.6b	2.9bc	1.3b	51.6e	29.4g	5.9b	6.8ab
	PF–2	3.3a	3.0a	68.6a	133.7a	NS	ND	2.1d	1.0d	56.3d	66.1f	3.8d	6.5b
	PF–2+NS	2.4c	1.7c	57.2b	71.5c	0.6c	6.0b	4.7a	1.8a	184.4a	237.2a	6.1b	7.4a
Mustang	Control	1.0d	0.8f	36.7h	4.3h	ND	ND	1.7f	0.9d	49.5f	36.6e	3.3e	6.0e
	HWG–1	3.2b	0.7g	41.8g	34.6e	2.7d	3.7d	2.1e	1.0d	48.3f	88.2c	6.2b	7.4c
	HWG–1+NS	2.1c	0.6h	54.9d	16.9g	2.7d	3.6d	2.8c	1.2b	60.7e	14.2g	6.0b	7.9b
	HWG–2	2.0c	2.3a	45.9f	96.0b	1.8e	4.7c	1.6fg	0.8e	87.5d	10.8g	4.4d	5.9e
	HWG–2+NS	2.1c	0.6h	72.7b	23.7f	ND	ND	3.8a	1.3a	42.6g	164.2b	4.0d	8.5b
	NS	0.8e	0.6i	49.5e	4.8h	3.9b	8.2a	1.5g	0.8e	142.9a	20.2f	4.2d	7.1c
	PF–1	2.2c	1.3d	52.0e	37.8d	4.9a	6.2b	1.2h	1.0cd	39.7h	168.0ab	5.0c	6.0e
	PF–1+NS	2.0c	0.9e	48.1f	18.9g	3.5c	4.7c	2.4d	1.1c	58.6d	165.0b	4.3d	7.0d
	PF–2	4.5a	1.9b	97.4a	144.8a	1.4f	4.1d	3.1b	1.0d	113.2b	52.8d	2.9f	7.0d
	PF–2+NS	2.7c	1.5c	66.2c	87.9c	3.5c	5.0c	3.5a	1.3a	105.0c	173.5a	9.5a	44.6a

N-Leaf: nitrogen content in leaf, N-Root: nitrogen content in root, TP-Leaf: total peptide in leaf, Suc-R: sucrose content in root, TS–R: total sugar content in root, RuBisCO: content of Ribulose-1,5-bisphosphate carboxylase/oxygenase, ND: not determined. Means are calculated from 3 replicates and separated using Turkey’s posthoc test at *p* < 0.05. Means followed by the same letter along the column are not significantly different. HWG: hydrolyzed wheat gluten, PF: potato protein film, -1: 1 g/kg, -2: 2 g/kg, +NS, in combination with nutrient solution.

**Table 2:** Correlation analysis of the effect of PBB treatment on sugar beet agronomic and physiological traits, with LNC below the diagonal and HNC above the diagonal.

		HNC												
		PH	CA	A	Gsw	CF	DSH	DRT	N-L	N-R	RuBisCO	Suc-R	TS–R	TP–L
LNC	PH	1.00	0.80 ***	−0.06	−0.13	0.19	0.67 ***	−0.15	0.09	0.12	0.08	−0.24	−0.14	0.14
	CA	0.87 ***	1.00	−0.15	−0.19	0.06	0.82 ***	0.30	−0.03	−0.07	0.18	0.03	−0.05	−0.07
	A	0.20	0.30	1.00	0.83 ***	−0.08	−0.01	−0.09	0.41 *	0.43 *	0.04	−0.03	0.01	0.44 *
	Gsw	−0.09	0.10	0.84 ***	1.00	−0.11	−0.09	0.06	0.31	0.27	0.12	0.10	−0.11	0.32
	CF	0.55 **	0.42 **	0.33	0.36 *	1.00	0.15	−0.39 *	−0.13	0.02	−0.10	−0.28	−0.06	−0.03
	DSH	0.87 ***	0.87 ***	0.56 **	0.37 *	0.52 **	1.00	0.31	0.24	0.15	0.21	0.06	0.08	0.14
	DRT	0.81 ***	0.77 ***	0.32	0.05	0.51 **	0.81 ***	1.00	−0.05	−0.29	0.17	0.46 *	0.06	−0.21
	N-L	0.62 ***	0.64 ***	0.53 **	0.50 ***	0.46 *	0.74 ***	0.45 *	1.00	0.84 ***	0.39 *	−0.02	0.25	0.91 ***
	N-R	0.22	0.31	0.55 **	0.67 ***	0.34	0.48 **	0.24	0.55 **	1.00	0.45 *	0.00	0.17	0.79 ***
	RuBisCo	0.37 *	0.43 *	0.47 **	0.61 ***	0.22	0.65 ***	0.28	0.72 ***	0.76 ***	1.00	0.47 **	0.33	0.26
	Suc-R	0.00	−0.22	−0.53 **	−0.54 **	−0.15	−0.19	−0.06	−0.31	−0.41*	−0.36	1.00	0.41 *	−0.32
	TS–R	0.36 *	0.35	−0.17	−0.14	0.03	0.31	0.36 *	0.10	0.18	0.02	0.40	1.00	0.09
	TP–L	0.41 *	0.48 **	0.40 *	0.52 **	0.49 **	0.61 ***	0.40 *	0.66 ***	0.82 ***	0.77 ***	−0.41 *	0.07	1.00

PH: plant height, CA: canopy area, A: photosynthetic assimilation rate, Gsw: stomata conductance, CF: chlorophyll fluorescence, DSH: shoot dry mass, DRT: root dry mass, N-L: nitrogen content in leaf, N-R: nitrogen content in root, RuBisCO: content of Ribulose-1,5-bisphosphate carboxylase/oxygenase Suc-R: sucrose content in root, TS–R: total sugar content in root, TP–L: total peptide in leaf. Values are correlation coefficient R, *: *p* < 0.05, **: *p* < 0.01 and ***: *p* < 0.001.

**Table 3:** Total of 24,255 CDS sequences were processed and used for Differentially Expressed Gene (DEG) analysis with two thresholds set. One is only on FDR < 0.05, and another one is on both FDR < 0.05 and Log2FoldcChange (Log2FC) > 1.0. The total number of genes DE for these two cut-offs is shown below. One with a stringent threshold (FDR < 0.05 and log2FC > 1) and another one without a stringent threshold (FDR < 0.05 and no log2FC cut-off was set). These data are available in Supplementary Table S2.

Comparisons	Leaf		Root	
	HWG vs. Control	PF vs. Control	HWG vs. Control	PF vs. Control
Total DE genes	14,320	14,193	15,258	15,280
Number of genes (P5e-2_C0)	4525	2437	7448	8441
Number of genes (P5e-2_C1)	906	409	1756	2693

P5e-2_C0: number of genes down-regulated, P5e-2_C1: number of genes upregulated.

**Table 4:** (**a**). Physical and chemical components of the peat-based medium used in the high nutrient condition (HNC) soil medium. (**b**). Content of major elements present in nutrient solution (NS).

(a)		
Serial Number	Description	Composition
1	Light peat	50%
2	Dark peat	33%
3	Gravel	7%
4	exclay/LWA (2–6 mm)	5%
5	Clay	5%
6	pH	5.5–6.5
7	EC	2.0–4.0
	Additional component	
1	Crushed limestone	6 kg
2	Dolomite lime	2 kg
3	NPK 11-5-18 & Trace elements	1.5 kg
4	Extra trace element	0.1 kg
5	Optifer	0.1 kg
**(b)**		
**Serial Number**	**Element**	**Concentration (mg/L)**
1	N	271
2	P	56
3	K	331
4	Mg	70
5	Ca	169
6	S	61

**Table 5:** Description of the growing media used in the experiment.

Factors	Levels	Remarks
Genotype	I (Volga), II (Armesa) and III (Mustang)	
Treatment name	Hydrolyzed wheat gluten	13.1% nitrogen present HWG
	Potato protein film	13.1% nitrogen present in PF
	Nutrient solution	NS contained major and minor nutrients, applied as a single solution and in one-time application (4 WAP). The solution contained 271 mg N per liter
Concentration (g/kg soil)	0, 1, 2	g of PBB per kg of soil
Treatments combinations	Control, HWG–1, HWG–2, HWG–1+NS, HWG–2+NS, NS, PF–1, PF–2, PF–1+NS, PF–2+NS	10 treatments per genotype

## Data Availability

The data are available in the Sequence Read Archive (SRA) database at NCBI under BioProject accession number PRJNA857348.

## Supplementary Materials

The supporting information can be downloaded at: https://www.mdpi.com/article/10.3390/ijms24119720/s1.
