# Supplementary material for: Metabolic Processes and Biological Macromolecules Defined the Positive Effects of Protein-Rich Biostimulants on Sugar Beet Plant Development
Source: Int J Mol Sci. 2023 Jun 3;24(11):9720. doi: 10.3390/ijms24119720 (PMC10253764; doi:10.3390/ijms24119720)
Supplement: Supplementary file 1 [file ijms-24-09720-s001.zip › ijms-2386715-Supplementary_Tabs and Figs.pdf]

Supplementary Table S1. Effects of PBB treatments on growth and physiological traits of sugar beet.

Trial	Treatment	Voga							Armesa							Mustang						
		PH	CA	DSH	DRT	A	Gsw	CF	PH	CA	DSH	DRT	A	Gsw	CF	PH	CA	DSH	DRT	A	Gsw	CF
Low nutrient content	Control	4.7g	0.5h	21.2f	55.9h	ND	ND	0.81a	3.4e	0.3e	29.7e	40.5h	ND	ND	0.79a	3.9f	0.3h	33.9g	61.4h	ND	ND	0.81a
	HWG-1	13.1c	16.7bc	946.5b	887.0a	13.6b	0.13d	0.84a	10.9b	8.6c	697.7c	316.2d	13.8c	0.11f	0.81a	12.6c	8.9d	788.2c	565.7bc	11.9c	0.05e	0.82a
	HWG-1+NS	15.1ab	15.2c	784.2c	576.8c	12.2c	0.10e	0.82a	14.0a	17.4a	957.5a	740.4a	16.9b	0.12e	0.83a	15.4a	15.3a	915.3b	579.8b	7.4e	0.03f	0.83a
	HWG-2	10.0e	8.8f	700.0d	276.8f	17.0a	0.22b	0.85a	8.8c	8.8bc	662.2c	173.0f	13.5c	0.13d	0.81a	12.4c	7.3e	997.4ab	522.2c	14.9a	0.13c	0.82a
	HWG-2+NS	16.1a	26.7a	1084.5a	420.0e	12.2c	0.10e	0.81a	11.7ab	15.3ab	944.9a	478.1c	15.5b	0.15c	0.81a	12.6c	8.5d	492.3e	357.8e	15.7a	0.12c	0.83a
	NS	7.2f	1.7g	108.0e	65.2g	4.8d	0.02f	0.82a	5.9d	0.9d	83.3d	121.7g	ND	ND	0.78a	6.1e	1.3g	82.7f	91.3g	2.0f	ND	0.80a
	PF-1	11.4d	11.5d	664.8d	474.6d	16.0a	0.24a	0.83a	11.3b	15.3ab	791.7b	655.7b	17.4ab	0.13e	0.81a	10.4d	6.2f	577.0d	294.6f	15.9a	0.15b	0.82a
	PF-1+NS	12.6cd	10.3e	802.7c	617.4c	12.1c	0.12d	0.82a	13.2ab	11.9b	795.0b	446.7c	13.7c	0.12e	0.82a	13.8b	12.4bc	760.6c	790.3a	13.7b	0.08d	0.82a
	PF-2	11.8d	17.4b	1174.1a	701.5b	16.3a	0.18c	0.81a	9.8c	7.9c	703.4c	276.7e	18.7a	0.24a	0.81a	12.4c	11.5c	1065.3a	531.7c	16.6a	0.22a	0.83a
	PF-2+NS	14.0b	17.6b	863.1bc	579.8c	13.9b	0.10e	0.82a	11.4bc	10.7b	854.3b	299.5de	17.0ab	0.19b	0.81a	14.9ab	13.5b	839.0c	456.5d	10.0d	0.04e	0.82a
	Mean	11.6b	12.6b	714.9b	465.5b	13.1b	0.13b	0.82a	10.0b	9.7b	652.0b	354.8b	15.8a	0.15b	0.81a	11.5b	8.5b	655.2b	425.1b	12b	0.09b	0.82a
High nutrient content	Control	21.2c	18.3c	1385.3c	658.8c	11.8e	0.14f	0.82a	17.6c	12.0d	1264.1d	866.7c	16.1c	0.44b	0.84a	20.5d	14.2f	1461.5e	753.5d	9.0e	0.10f	0.83a
	HWG-1	27.0ab	19.3bc	1336.2c	240.0h	14.9cd	0.34b	0.82a	22.8b	20.3c	1590.8c	651.3e	19.2ab	0.35c	0.83a	25.8b	30.8bc	2232.7b	921.2c	8.7e	0.09g	0.84a
	HWG-1+NS	27.5a	33.9a	2268.1a	781.3b	14.2d	0.22e	0.84a	23.1b	27.1a	1742.6c	1077.9b	14.5d	0.28e	0.82a	22.7c	18.5e	1638.5d	762.9d	20.0a	0.37a	0.84a
	HWG-2	26.0ab	31.4ab	2230.7a	629.7cd	16.3c	0.22e	0.84a	23.3b	27.8a	2284.4a	1612.1a	21.2a	0.52a	0.81a	29.8a	42.6a	2549.9a	886.4b	10.3d	0.11e	0.84a
	HWG-2+NS	27.0a	25.6b	2295.2a	520.0e	18.3b	0.30c	0.84a	26.0a	25.9ab	1884.1bc	679.9e	18.2b	0.43b	0.83a	24.5bc	23.3d	2041.3bc	751.6de	12.4c	0.39a	0.84a
	NS	22.0c	22.1b	1647.8b	857.2a	14.0d	0.21e	0.84a	21.6b	19.5c	1330.0d	687.3e	14.3d	0.23f	0.82a	22.1c	21.2d	1592.2de	1127.2b	9.7d	0.11e	0.83a
	PF-1	23.1b	19.3bc	1649.9b	474.9f	20.7a	0.39a	0.83a	22.0b	19.0c	1924.8b	811.2d	12.8e	0.16h	0.82a	28.8a	33.2b	1932.2c	756.9de	14.7b	0.27b	0.83a
	PF-1+NS	24.0b	19.2bc	1568.3b	326.4g	15.9c	0.26d	0.84a	24.5ab	26.7ab	2058.1b	786.7d	15.6cd	0.27e	0.82a	26.0b	25.8cd	2149.6b	717.8e	12.2c	0.16d	0.84a
	PF-2	24.5b	19.3bc	1660.0b	322.1g	16.2c	0.25d	0.83a	23.8b	25.1b	1590.8c	496.6f	12.6e	0.19g	0.83a	25.9b	27.8c	1927.0c	1448.2a	12.8c	0.20c	0.82a
	PF-2+NS	26.0ab	24.3b	2090.1a	591.7d	16.6c	0.24d	0.83a	22.5b	27.2ab	1921.4b	1107.5b	17.1b	0.32d	0.82a	22.2c	21.6d	1953.7c	816.5d	15.3b	0.21c	0.83a
	Mean	24.8a	23.3a	1813.1a	540.2a	15.9a	0.26a	0.83a	22.7a	23.1a	1759.1a	877.7a	16.2a	0.32a	0.82a	24.8a	25.9a	1947.8a	894.2a	12.5a	0.20a	0.83a

PH- plant height, CA- plant canopy area, DSH- shoot dry mass, DRT- root dry weight, A- photosynthetic carbon assimilation, Gsw- stomata conductance, CF- chlorophyll fluorescence, ND- not determined, due to limited sample. Means are calculated from 5 replicates and separated using Tukey's posthoc test at $p<0.05$. Means followed by similar letters along the column are not significantly different.

Supplementary Table S2. Initial filtered reads in millions, total mapped and un-mapped reads to the reference genome in percentage. Sample IDs, sample short and full names described here were used in the differential gene expression (DGE) analysis.

Sample ID	Sample short Name	Sample Full Name	Before filter (M Seqs)	After filter (M Seqs)	Uniquely mapped reads %	Number of reads mapped to multiple loci %	Number of reads mapped to too many loci%	Number of reads unmapped: too short%	Number of reads unmapped: other%
P21526_128	LfCl1	Leaf-Control1	18.9	**15.0**	**87.95%**	3.25%	0.67%	6.91%	1.22%
P21526_129	LfCl2	Leaf-Control2	20.6	**15.2**	**88.16%**	3.30%	0.65%	6.78%	1.11%
P21526_130	LfCl3	Leaf-Control3	20.9	**17.3**	**88.84%**	3.28%	0.63%	6.11%	1.14%
P21526_131	LfWt1	Leaf-Wheat1	48.5	**33.6**	**85.63%**	2.79%	0.24%	10.80%	0.53%
P21526_132	LfWt2	Leaf-Wheat2	48.5	**33.6**	**85.63%**	2.79%	0.24%	10.80%	0.53%
P21526_133	LfWt3	Leaf-Wheat3	22.4	**16.2**	**89.44%**	2.78%	0.23%	6.99%	0.57%
P21526_134	LfPo1	Leaf-Potato1	23.0	**17.6**	**87.95%**	3.47%	0.63%	6.94%	1.01%
P21526_135	LfPo2	Leaf-Potato2	55.4	**41.4**	**88.89%**	3.47%	0.57%	5.90%	1.17%
P21526_136	LfPo3	Leaf-Potato3	24.7	**19.1**	**89.92%**	3.52%	0.62%	4.81%	1.13%
P21526_137	RtCl1	Root-Control1	16.6	**13.1**	**87.27%**	3.10%	0.45%	8.38%	0.79%
P21526_138	RtCl2	Root-Control2	58.4	**47.1**	**88.22%**	3.33%	0.42%	7.24%	0.78%
P21526_139	RtCl3	Root-Control3	21.1	**17.9**	**87.94%**	3.26%	0.43%	7.65%	0.72%
P21526_140	RtWt1	Root-Wheat1	25.2	**21.5**	**87.82%**	3.87%	0.63%	6.51%	1.18%
P21526_141	RtWt2	Root-Wheat2	60.6	**44.7**	**86.65%**	3.88%	0.66%	7.56%	1.24%
P21526_142	RtWt3	Root-Wheat3	26.9	**20.3**	**86.33%**	3.90%	0.66%	7.93%	1.18%
P21526_143	RtPo1	Root-Potato1	23.6	**17.7**	**87.44%**	4.16%	1.04%	5.96%	1.41%
P21526_144	RtPo2	Root-Potato2	19.0	**14.7**	**88.83%**	4.04%	1.00%	4.63%	1.51%
P21526_145	RtPo3	Root-Potato3	21.8	**16.1**	**86.56%**	4.13%	1.05%	6.97%	1.29%

Supplementary Table S3. List of all differentially expressed genes (DEGs) (from Table 1) from all pairwise (LfPo-vs-LfCl, LfWt-vs-LfCl, RtPo-vs-RtCl, RtWt-vs-RtCl) and group (Po-vs-Cl, Wt-vs-Cl) genotype comparisons. The table contains the expression values (estimated raw read counts) in three biological replicates, with cut-off FDR<5e-2 (P5e-2) and no log2fold change cut-off was set (C0), and the functional annotations of genes were included.

Supplementary Table S4. Results from KEGG pathway enrichment analysis based on the list of differentially expressed transcripts (DEGs) in both pairwise (Supplementary Figure S1) and group comparisons, The DE gene set filtered cut-off was set to FDR < 0.05.

Supplementary Table S5. Gene ontology (GO) term enrichment analysis was performed on the DE gene set filtered with FDR < 0.05. The total of six comparisons both pairwise and group comparisons.

Supplementary Table S6. List of primers used in the study

Primer name	Primer sequence
BvGAPDHqPCR.F	CACCACCGATTACATGACATACA
BvGAPDHqPCR7R	GGATCTCCTCTGGGTTCCTG
EF1AlphaqPCR7F	GCTTTTGAGGATCTCTGGCG
EF1Alpha qPCR7R	AAGCCTTAGAGTCAGCTGCT
HSP70F	GTCAGGCTACCAAGGATGCT
HSP70R	TCTTGTCCAGACCATAGGCA
GR2.6F	ACAATCGGCGATCTTGATGC
GR2.6R	CCGGCAAACAAGATCCACAA
HIPP24F	CAACAGAAGGTGACGGTGAC
HIPP24R	GCACATAAGGCCATAACTCCA
IAA6F	GGCCACACCGAGAAAGTATC
IAA6R	TTACCAATGCTCCTCCTCCT
SUSIBA2F	CTGCCATGTTCAAGCCTTGT
SUSIBA2R	ATTCGCTGCCAAATCACCAG

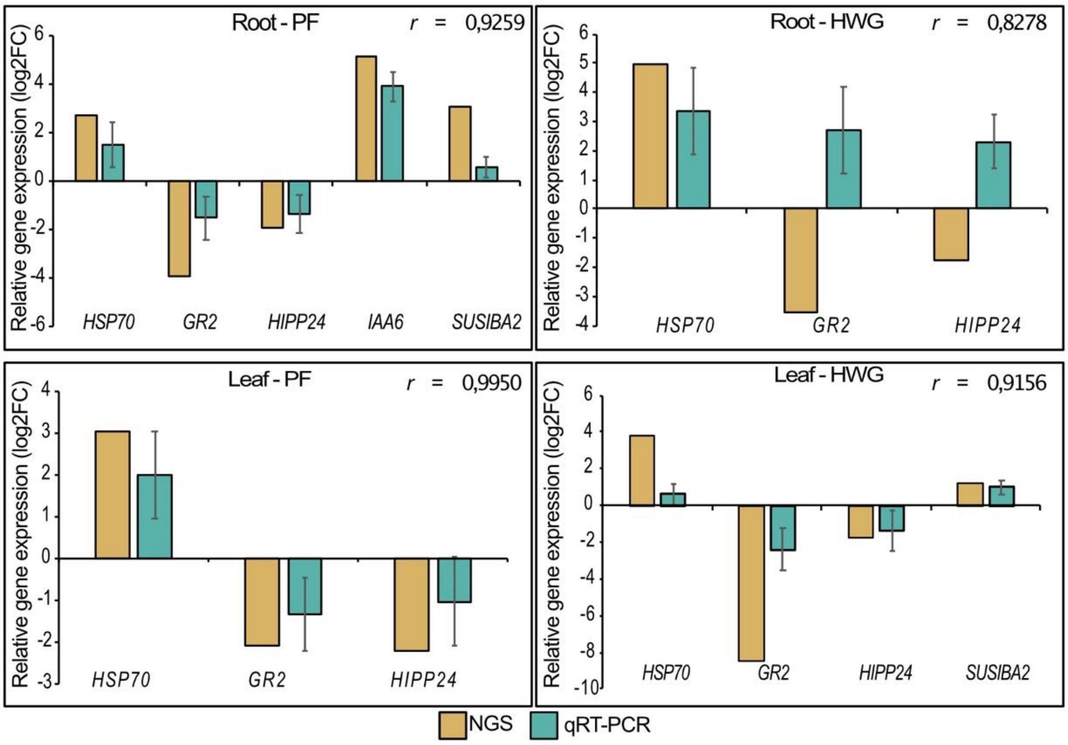

Supplementary Figure S1. Relative expression of *BvHSP70*, *BvGR2*, *BvHIPP24*, *BvIAA6,* and *BvSUSIBA2* in roots and leaves of sugar beet plants treated with HWG and PF.

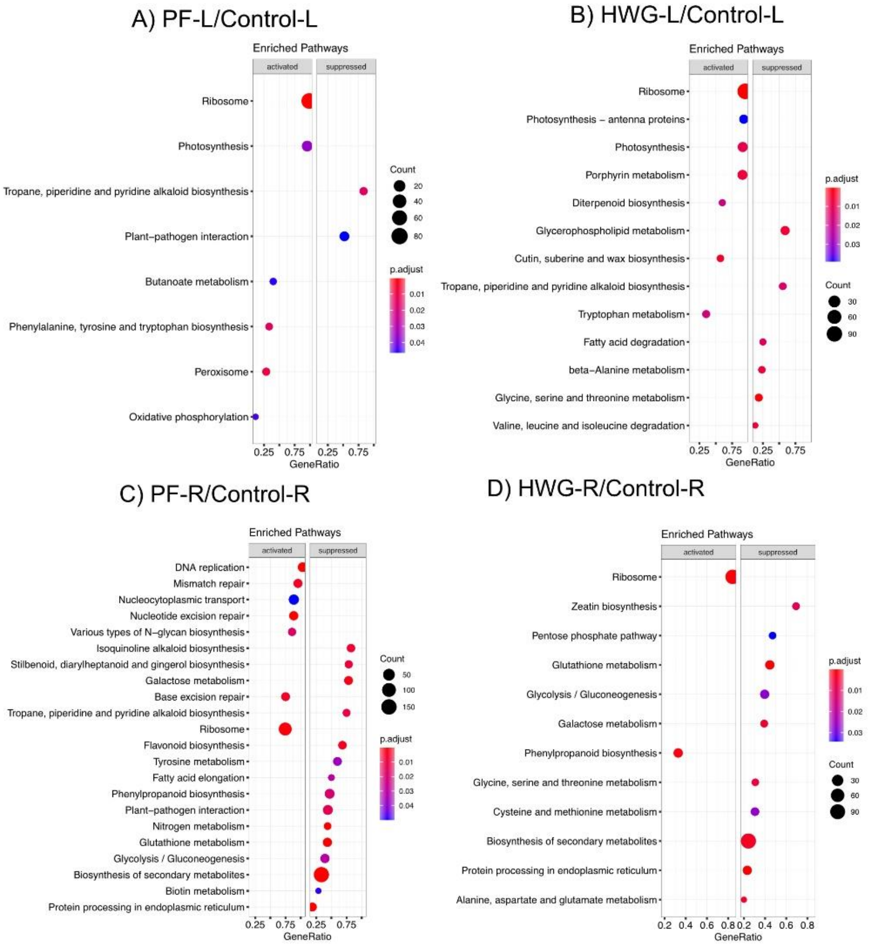

Supplementary Figure S2. Bubble plots of KEGG pathway enrichment analysis from pairwise comparisons (PF-L/Control-L, HWG-L/Control-L, PF-R/Control-R and HWG-R/Control-R) of DEGs, complete data was shown in Supplementary Table 4. **(A).** PF-L/Control-L: leaf sample treatment with potato protein film (PF) compared to leaf sample of control treatment. **(B).** HWG-L/Control-L: leaf sample treatment with hydrolysed wheat gluten (HWG) compared to leaf sample of control treatment. **(C).** PF-R/Control-R: root sample treatment with potato protein film (PF) compared to root sample of control treatment. **(D).** HWG-R/Control-R: root sample treatment with hydrolysed wheat gluten (HWG) compared to root sample of control treatment
